# Phytochemical Composition and Wound Healing Properties of *Echinacea angustifolia* DC. Root Hydroalcoholic Extract

**DOI:** 10.3390/ijms26062562

**Published:** 2025-03-12

**Authors:** Daniela Russo, Ludovica Lela, Nadia Benedetto, Immacolata Faraone, Gianluca Paternoster, Patricia Valentão, Luigi Milella, Monica Carmosino

**Affiliations:** 1 Department of Health Science, University of Basilicata, Via dell’Ateneo Lucano 10, 85100 Potenza, Italy; daniela.russo@unibas.it (D.R.); ludovica.lela@unibas.it (L.L.); nadia.benedetto@unibas.it (N.B.); immacolata.faraone@unibas.it (I.F.); gianluca.paternoster@ospedalesancarlo.it (G.P.); monica.carmosino@unibas.it (M.C.); 2 REQUIMTE/LAQV, Laboratório de Farmacognosia, Departamento de Química, Faculdade de Farmácia, Universidade do Porto, R. Jorge Viterbo Ferreira, n° 228, 4050-313 Porto, Portugal; valentao@ff.up.pt

**Keywords:** *Echinacea angustifolia*, antioxidants, anti-inflammatory, wound healing, root extract

## Abstract

The therapeutic potential of natural products has led to the exploitation of phytocomplexes for treating various skin conditions, including wounds. *Echinacea angustifolia* DC. has traditionally been used for wound healing, burns, and other ailments. In this study, dried roots of *E. angustifolia* were extracted using a hydroalcoholic solution, and the phytochemical composition was analyzed through HPLC-DAD. The polyphenol and polysaccharide content, along with in vitro antioxidant and anti-tyrosinase properties, were evaluated. The biological effect of *E. angustifolia* extract was evaluated on the 3T3-L1 cell line. HPLC-DAD analysis confirmed the presence of several polyphenols, particularly caffeic acid derivatives, with echinacoside as the predominant compound, exhibiting strong antioxidant properties. The extract demonstrated no cytotoxic effect on 3T3-L1 cells, and it showed a protective effect by increasing the migration process in an in vitro scratch wound healing test, together with echinacoside and allantoin, which were used as references. Furthermore, the extract reduced the expression of proinflammatory cytokines and promoted that of proteins that accelerate wound closure, such as TGF*-β*1. The present study demonstrates the potential wound healing properties and the antioxidant and anti-inflammatory activity of *E. angustifolia* root hydroalcoholic extract, giving a scientific rationale for its traditional use.

## 1. Introduction

The wound-healing process is a dynamic and complex biological response that involves cellular, molecular, and biochemical interactions, as well as physiological activities, to regenerate and replace damaged connective tissue [1]. This process consists of several key phases, including hemostasis, inflammation, oxidative stress response, proliferation, migration, and tissue maturation [2]. Endothelial and fibroblast cells play a crucial role in the reparative phase of the skin. These cells are recruited to the wound site by inflammatory mediators, where they proliferate and migrate to fill the tissue gap and restore the skin barrier (proliferation and migration phase). Fibroblasts are involved in the production of several components, such as collagen, one the most important components of the extracellular matrix of the skin. Subsequently, the remodeling and maturation of the tissue allow the full recovery of the skin tissue, sometimes leaving no traces of skin damage. Fibroblasts then participate in the construction of scar tissue and its remodeling [3].

There is growing interest in natural compounds as complementary therapies for managing skin conditions, wounds, inflammation, etc. [4,5]. Treatment of wounds is frequently studied in ethnopharmacological research [3,6,7]. Numerous plant species have been investigated for their potential to accelerate wound healing [8,9,10]. However, in many cases, scientific validation of their efficacy remains limited, and knowledge of their chemical composition and mechanisms of action is incomplete.

The *Echinacea* genus (Asteraceae family) comprises nine species, of which three are commercially used: *E. angustifolia* DC., *E. purpurea* L., and *E. pallida* Nutt. [11]. Traditionally, these North American species have been used to treat wounds, snakebites, and burns, as well as various ailments such as toothaches, coughs, and colds. The first documented commercial use of *Echinacea* dates back to the early 20th century, when hydroalcoholic extracts were introduced to the market [12]. Today, *Echinacea* remains an important commercial botanical due to its widespread use and well-documented pharmacological properties [13].

The phytochemical composition of *Echinacea* is well documented, and three main classes of bioactive compounds (alkamides, polysaccharides, and polyphenols) are considered responsible for its biological effects [14,15,16,17,18]. The most important identified polyphenols are caffeic acid and its derivatives, such as echinacoside, verbascoside, and cynarin, together with chlorogenic, caftaric, and chicoric acids [16].

*Echinacea* species are known for their anti-inflammatory and immunostimulant properties and have been traditionally used in phytotherapy for wound healing [19,20]. *E. pallida* was tested to evaluate its wound-healing effect in mice by oral administration of the plant extract [21]. Additionally, previous research has reported the anti-inflammatory and cicatrizing properties of gel formulations containing echinacoside, as well as *E. pallida* and *E. purpurea* root extracts, in rats with skin abrasions and excision wounds [22]. It is well known that *Echinacea* species exhibit differences in chemical composition, which influence their biological activity. In fact, *E. purpurea* is rich in chicoric acid, but echinacoside is absent. Echinacoside is abundant in *E. angustifolia*, along with cynarin, and *E. pallida* contains low levels of alkamides [18]. To the best of our knowledge, the wound-healing effect of *E. angustifolia* root extract has not yet been reported.

Therefore, this study aimed to evaluate the effect of the hydroalcoholic root extract of *E. angustifolia* in terms of the biological properties and the proliferation and migration capacity using an in vitro wound assay. The latter can be considered a valuable and inexpensive tool for investigating the wound-healing properties of natural compounds [1,23].

## 2. Results

### 2.1. Phytochemical Composition

Chemical analysis of *Echinacea* has reported the presence of phenolic compounds (caffeic acid derivatives), polysaccharides, and alkilamides [16]. In our study, *E. angustifolia* roots were extracted using an 85% ethanol solution, giving an extraction yield—which represents the weight of the extract per 100g of raw material of 2.60%. *E. angustifolia* root extract (EAR) showed a polysaccharide content of 17.32 ± 1.33 mg Glu/g DW.

Then, the Folin–Ciocalteu reagent was used to determine the content of polyphenols; the EAR extract showed a value of 50.36 ± 3.99 mg GAE/g of dried extract.

**Figure 1:**
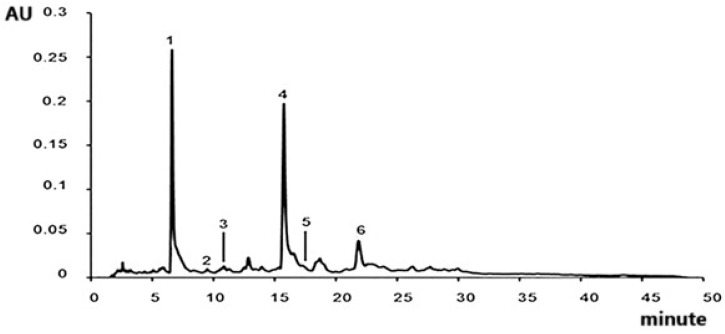
Chromatogram representative of all identified compounds in roots of *E. angustifolia* extract at 320 nm: caftaric acid (1), chlorogenic acid (2), caffeic acid (3), echinacoside (4), ferulic acid (5), and verbascoside (6).

Qualitative analysis of EAR was performed using HPLC-DAD and by comparison of the retention time and UV–Vis spectrum of standards. Caftaric acid (peak 1, Rt: 7.311 min), chlorogenic acid (peak 2, Rt: 8.252 min), caffeic acid (peak 3, Rt: 8.715 min), echinacoside (peak 4, Rt: 9.702 min), ferulic acid (peak 5, Rt: 13.429 min), and verbascoside (peak 6, Rt: 15.928 min) were identified in the roots of *E. angustifolia* (Figure 1).

The compounds identified by HPLC-DAD were confirmed by LC-MS/MS analysis as reported in Table 1.

**Figure 2:**
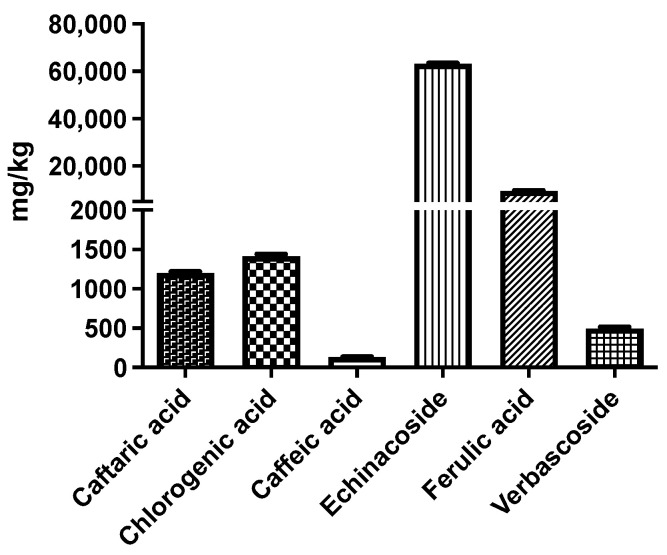
HPLC-DAD quantification of identified compounds from *E. angustifolia* root extract; results are expressed as mg per kg of dried roots.

Based on the quantitative HPLC analysis (Figure 2), echinacoside is the most representative compound in EAR extract. In fact, it is the most abundant among the identified compounds (63181.24 ± 273.90 mg echinacoside/kg of extract). A high content of ferulic acid (9467.59 ± 199.33 mg ferulic acid/kg of extract), chlorogenic acid (1414.05 ± 26.06 mg chlorogenic acid/kg of extract), and caftaric acid (1201.72 ± 17.71 mg caftaric acid/kg of extract) were also observed.

### 2.2. Cell Viability and Proliferation of 3T3 L1 Cells

**Figure 3:**
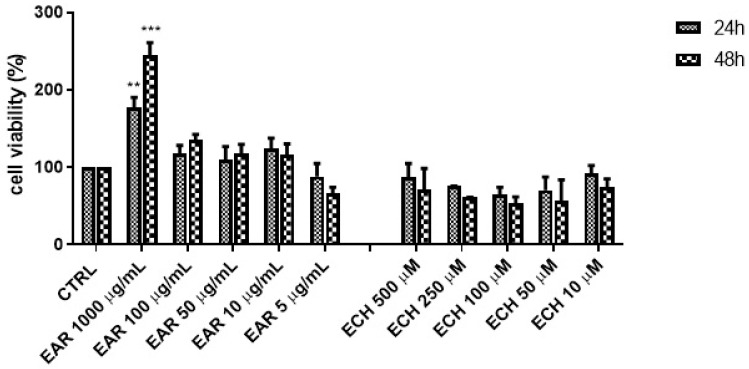
3T3-L1 cell viability by calcein-AM assay of *E. angustifolia* root extract (EAR) and echinacoside standard; ** *p* < 0.01, *** *p* < 0.001 vs. control cells (CTRL).

The calcein-based cell viability method was conducted to investigate the effect of several concentrations of EAR on 3T3-L1 viability/toxicity at both 24 h and 48 h (Figure 3).

The wound-healing effects of EAR were compared to those of echinacoside, the main component of EAR, as reported by quantitative HPLC analysis. Notably, the highest extract concentration (1000 μg/mL) significantly enhanced the cell proliferation rate, increasing it approximately threefold compared to the control. However, at lower concentrations (5–100 μg/mL), the proliferation rate remained statistically indistinguishable from that of untreated cells (CTRL).

Echinacoside was found to be safe and not toxic within the tested concentration range (0.01–0.50 mM), although it did not stimulate cell proliferation.

### 2.3. Hydrogen Peroxide-Induced Stress

**Figure 4:**
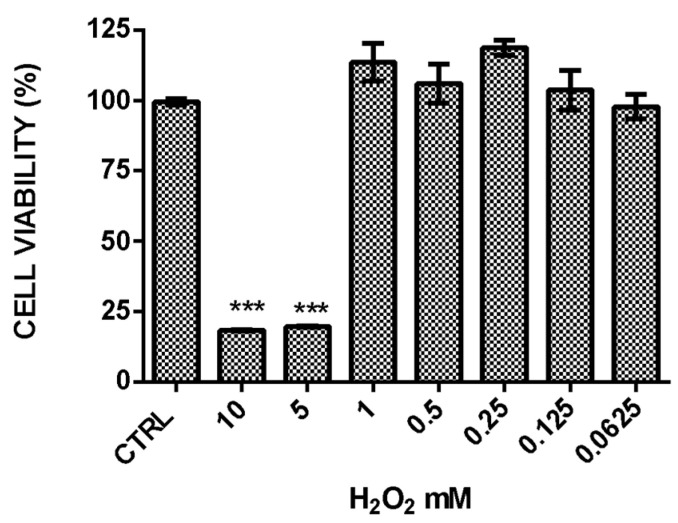
The curve with H_2_O_2_ concentrations using calcein-AM. Data are expressed as mean ± SD (*n* = 9). *** indicates *p* < 0.001 vs. H_2_O_2_ CTRL.

To assess the protective effects of the extract against oxidative stress, 3T3-L1 fibroblast cells were treated with hydrogen peroxide. A dose–response curve was generated to determine the optimal H_2_O_2_ concentration for the experiment (Figure 4).

**Figure 5:**
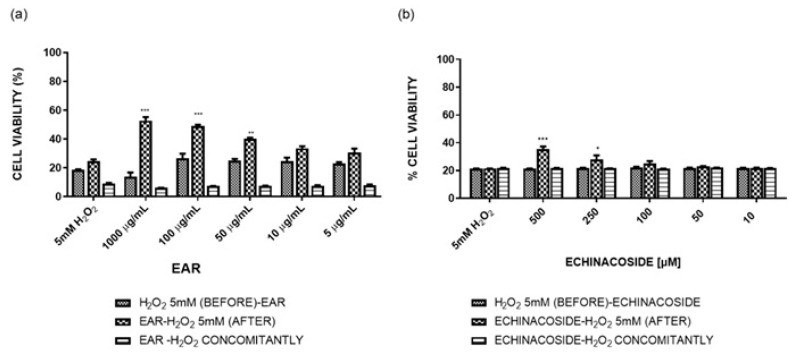
Viability (%) of cells treated with *Echinacea angustifolia* root extract (EAR) (**a**) and echinacoside (**b**) before, concomitantly, and after H_2_O_2_ exposure. Data are expressed as a mean ± SD (*n* = 9). * indicates *p* < 0.01, ** indicates *p* < 0.05, *** indicates *p* < 0.005 vs. H_2_O_2_ control (GraphPad Prism 5 Software).

A concentration of 5 mM H_2_O_2_ was selected for the experiments, as it reduced the cell viability by 80% after 24 h of exposure, as measured using the calcein-AM reagent. The antioxidant ability of EAR was evaluated before and after the exposure to H_2_O_2_ (5 mM) for 3 h and concomitantly for 24 h. The results showed that 3T3-L1 cells were not protected against oxidative stress when extract treatment was carried out before or concomitantly with H_2_O_2_, showing a low cell survival rate. However, a protective effect against H_2_O_2_-induced oxidative stress was observed when cells were pretreated with higher concentrations of EAR (50, 100, and 1000 μg/mL) for 24 h, preserving cell viability (Figure 5a). Echinacoside exhibited a similar trend to the extract, providing protection against H_2_O_2_-induced oxidative stress (Figure 5b). Notably, at its highest concentration (0.5 mM), echinacoside nearly doubled the cell viability compared to the untreated cells.

### 2.4. Antioxidant and Wound Healing Properties

**Figure 6:**
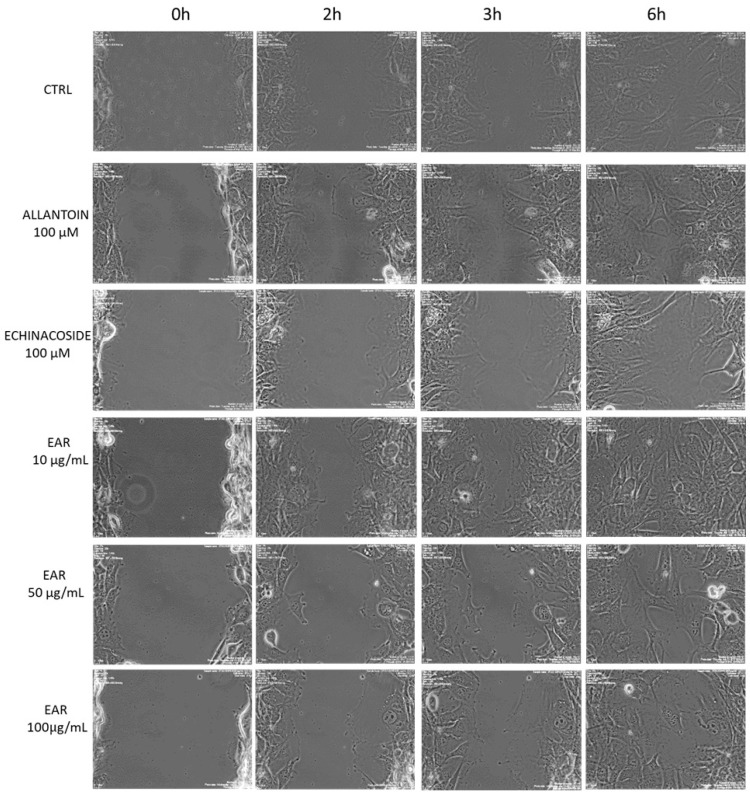
Microscopy images of 3T3L1 migration after scratch. CTRL, control; EAR, *E. angustifolia* root extract. Pictures were acquired by using the following parameters: filter Ph, objective 20×, light 19, exposure time 1/40 s, gain 1.00, resolution 800 × 600 binning.

Wound healing was simulated using an in vitro scratch assay, which involved disrupting the 3T3-L1 cell monolayer. The loss of contact between cells leads to the release of cytokines and growth factors near the wound, promoting cell migration and proliferation. Cell proliferation and migration have been found to be important factors for the tissue formation phase during wound healing. *E. angustifolia* extract was tested on the 3T3-L1 fibroblast cell line to evaluate its ability to stimulate cell migration by in vitro scratch assay. Allantoin (100 μM) was used as the positive control. The 3T3-L1 cells showed a fast migration rate by rapidly closing the wound (Figure 6).

**Figure 7:**
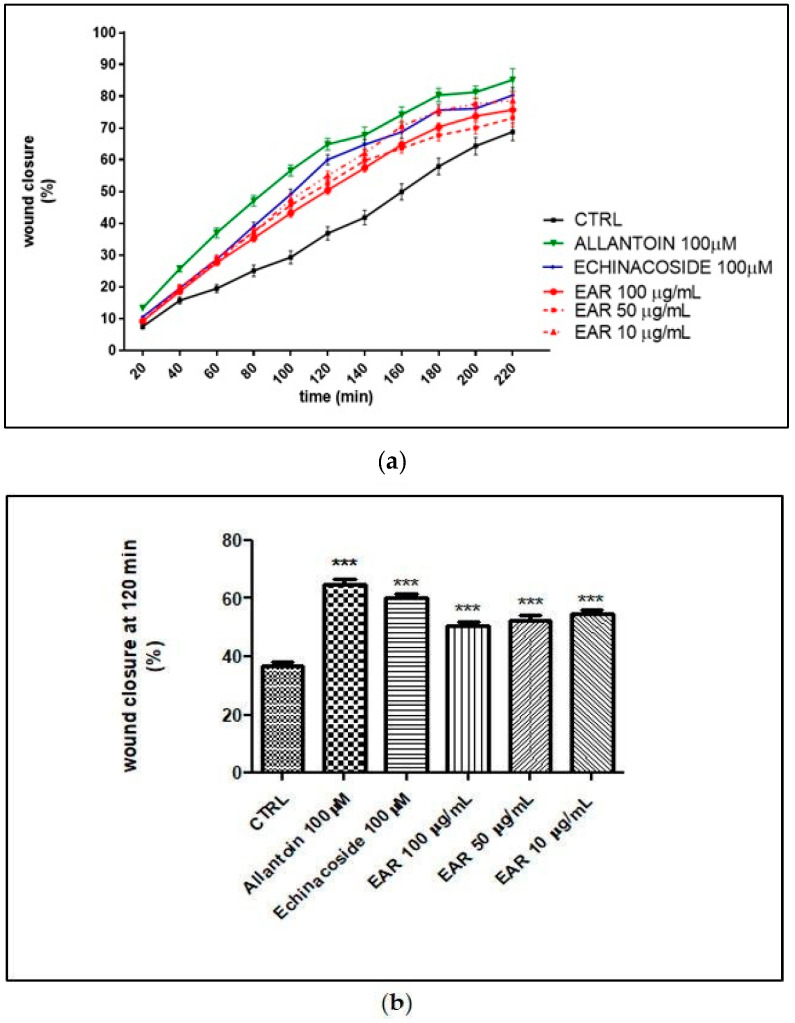
Wound healing rate over time (**a**) and at 120 min (**b**). *** indicates *p* < 0.005 against control. CTRL, control; EAR, *E. angustifolia* root extract.

The extract concentrations of 10, 50, and 100 μg/mL significantly increased the 3T3-L1 migration rate compared to the control (Figure 7).

In addition, echinacoside and allantoin significantly increased the rate of cell migration at the tested concentration (Figure 7). Interestingly, after 120 min of treatment, the percentage of wound closure was 36.82 ± 1.25% for the control, 64.85 ± 1.91% for allantoin (100 μM), 60.01 ± 1.59% for echinacoside (100 μM), and 50.40 ± 1.36%, 52.56 ± 1.55%, and 54.97 ± 1.43% for EAR at 100, 50, and 10 μg/mL, respectively. EAR significantly increased the rate of cell migration compared to the control and compared to echinacoside and allantoin, even at the lowest concentration tested.

Moreover, the *E. angustifolia* root extract reported a very low inhibition of the tyrosinase enzyme, compared to the reference, kojic acid (IC_50_ = 27.77 ± 3.41 μg/mL). The extract showed an inhibition of 7.52 ± 0.70% at 5 mg/mL.

**Figure 8:**
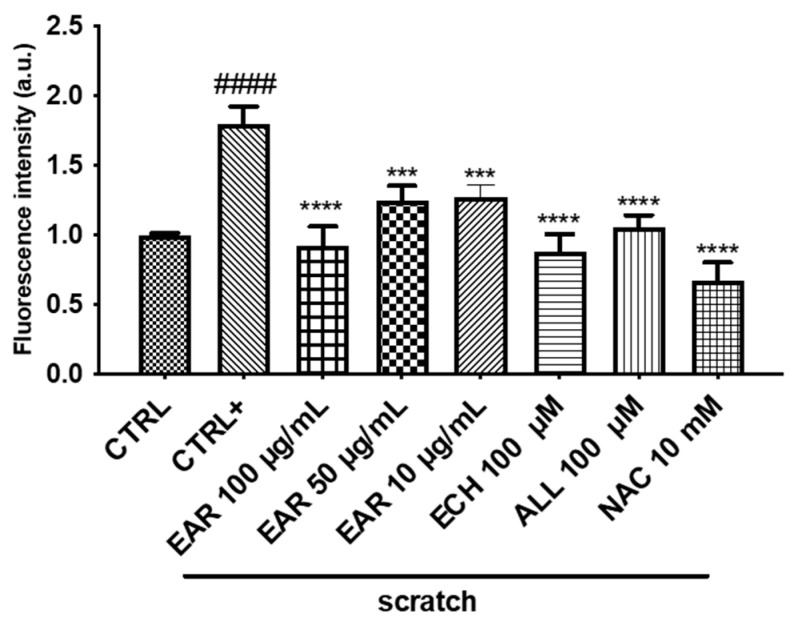
Effect of *E. angustifolia* root extract (EAR) on intracellular ROS generation in 3T3-L1 cells under scratch stress conditions. Data are expressed as the mean ± SD of three independent experiments (*n* = 3) and were analyzed by one-way ANOVA followed by Tukey’s post-hoc test. #### *p* < 0.001 vs. CTRL, **** *p* < 0.0001, *** *p* < 0.001 vs. scratched cells (CTRL +).

The wound-healing process is often accompanied by a reduction in oxidative stress. The antioxidant activity was initially examined by spectrophotometric assays, which reported a lipid peroxidation inhibition of 50.24 ± 1.38% (at 250 μg/mL) and DPPH scavenging activity of 243.24 ± 4.33 mg TE/g (IC_50_ = 36.79 ± 0.66 μg/mL) of dry extract. Subsequently, the EAR treatment showed a protective effect from oxidative stress in a dose-dependent manner, reducing the ROS levels by approximately 50% compared to untreated scratched 3T3-L1 cells (CTRL+). Furthermore, as shown in Figure 8, echinacoside, allantoin, and NAC (an antioxidant agent) statistically reduced the ROS to baseline levels.

### 2.5. Anti-Inflammatory Gene and Protein Expression

**Figure 9:**
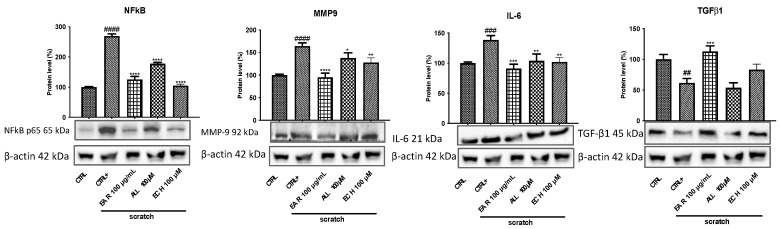
Effect of *E. angustifolia* root extract (EAR) on anti-inflammatory markers’ expression in 3T3-L1 cells after 6 h of treatment in stressed conditions. Densitometric analysis of the immunoreactive bands are expressed as the mean ± SD of three independent experiments (*n* = 3). The protein levels were normalized with β-actin content. Data were normalized to control cells set to 100%. #### *p* < 0.0001, ### *p* < 0.001, and ## *p* < 0.01 vs. control cells (CTRL); **** *p* < 0.0001, *** *p* < 0.001, ** *p* < 0.01, and * *p* < 0.05 vs. scratched cells (CTRL+).

To define the mechanism of action of the extract and the targets involved in the inflammatory status, the expression of MMP9, NF-κB, IL-6, and TGF-*β*1 proteins was evaluated by Western blot, while the gene expression of interleukins *TNF-α* and *IL-*1*β* were evaluated by qRT-PCR. Cells affected by scratch (CTRL+) showed a significant increase in MMP9, IL-6, and NF-κB protein expression compared to cells without scratching. Treatment with EAR (100 μg/mL), allantoin (100 μM), and echinacoside (100 μM) reduced these levels in all the proteins considered (Figure 9). Regarding TGF-*β*1, it is a cytokine highly important for successful wound closure. Its levels are reduced in scratched cell stress. EAR treatment increased the protein expression; however, echinacoside and allantoin showed no significant effects.

**Figure 10:**
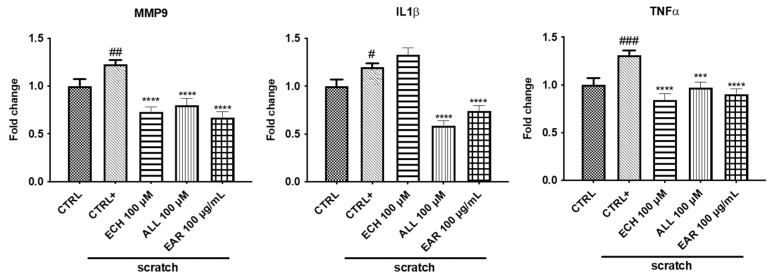
Effects of *E. angustifolia* root extract (EAR) on gene expression. The scratches increased the expression of *MMP9*, *TNF-α,* and *IL-1β*. EAR, allantoin, and echinacoside treatment reduced their levels. Data are normalized with the housekeeping gene polymerase II (Pol II) and are shown as fold change treated/control ± standard deviation (SD). ### *p* < 0.001, ## *p* < 0.01, and # *p* < 0.05 vs. control cells (CTRL); **** *p*< 0.0001 and *** *p* < 0.001 vs. scratched cells (CTRL+).

By qRT-PCR, the gene expression of the proinflammatory cytokines *TNF-α, IL-1β*, and *MMP-9* was increased in the stressed cells compared to the control cells (Figure 10). EAR treatment statistically decreased the expression of all genes considered, together with allantoin. As for echinacoside, it did not show any significant effect on *IL-1β*.

## 3. Discussion

This study investigated the antioxidant and anti-inflammatory properties and wound healing capacity of the hydroalcoholic root extract of *E. angustifolia* on 3T3-L1 cell lines.

Injuries occur due to soft tissue disruption, while the regulation of inflammation and oxidation plays an important role in wound healing by restoring cellular structures. High free radical production in cutaneous damaged areas can affect the healing process by compromising the physiological function and chemistry of proteins, lipids, and extracellular matrix (ECM) elements. Several medicinal herbs have recently been reported for in vivo and in vitro wound healing effects using preclinical models [30]. Active phytochemicals such as antioxidants and free radical scavengers of plant extracts may be involved in the wound healing process [31].

As with any extraction technique, the solvent plays an important role in the recovery of bioactive compounds. Previous studies [16,17] have reported that organic solvents, ethanol or methanol, are preferred to other solvents for extracting phenolic compounds from *Echinacea* species. Pellati et al., 2025 [16] reported that the most efficient solution for *Echinacea’s* compounds extraction ranged from 80 to 90% of organic solvent. In addition to solvent, applying different aqueous extraction techniques of *E. angustifolia* roots showed important differences in terms of the composition and quantity of the chemical substances recovered [32].

*Echinacea* is a rich source of polysaccharides; however, using hydroalcoholic solutions for extraction significantly reduces the yield of these compounds. In contrast, aqueous extracts [32] exhibit an average polysaccharide content approximately seven times higher than that of the EAR. It is well known that *E. angustifolia* root is also a valuable source of polyphenols, which are more efficiently extracted using hydroalcoholic solvents. Indeed, the results obtained in this study were higher than those reported in previous research involving aqueous extracts [32]. The polyphenol content has also been assessed in other *Echinacea* species, such as *E. purpurea,* reporting TPC values ranging from 11.00 ± 1.00 mg GAE/g to 60.00 ± 1.00 mg GAE/g [33].

The compounds identified by HPLC-DAD in the present investigation had already been detected in the roots of *E. angustifolia* [32]. However, the hydroalcoholic mixture did not enable the recovery of 3,5- and 3,4-dicaffeoylquinic acids, likely due to their higher solubility in aqueous solvents resulting from their acidic functional groups [32].

Furthermore, the hydroalcoholic extract obtained in our study exhibited higher antioxidant properties than aqueous extracts [32]. Russo et al. [32] reported that aqueous extracts displayed DPPH scavenging activity ranging from 14.63 ± 0.29 to 80.56 ± 4.52 mg TE/g and lipid peroxidation inhibition ranging from 9.07 ± 1.32 to 36.76 ± 4.22%. Conversely, Pellati et al. [16] evaluated the methanolic extract of three *Echinacea* species, reporting average IC_50_ values against the DPPH radical of 139.00 ± 0.90, 249.00 ± 2.50, and 211.00 ± 1.90 μg/mL for *E. purpurea*, *E. pallida,* and *E. angustifolia*, respectively. The mother tincture of *E. angustifolia,* prepared with 55% ethanol, was found to be less active than the hydroalcoholic extract tested in this study [34]. Specifically, it exhibited a DPPH value of 131.33 ± 14.26 mg TE/100 mL of tincture and an inhibition of lipid peroxidation, measured by the BCB test, of approximately 25%. Additionally, *E. purpurea* flower extract has been reported to possess notable antioxidant properties [35]. The high antioxidant activity observed in *E. angustifolia* may be attributed to echinacoside, the main compound of EAR. To date, both in vitro and in vivo studies have demonstrated the strong antioxidant potential of echinacoside [36]. Furthermore, minor compounds present in the extract also contribute to antioxidant activity, reinforcing the significant antioxidant properties of EAR [37,38,39].

Cell cultures are widely recognized as effective models for assessing the biological activity of plant extracts. In particular, fibroblasts, including the 3T3-L1 cell line, have been proposed as a suitable in vitro model for evaluating wound-healing potential [40].

In this study, the highest concentration of EAR significantly enhanced 3T3-L1 cell proliferation. Notably, no toxic effects were reported for *E. purpurea* hydroalcoholic extract on other cell lines, such as peripheral blood mononuclear cells (PBMCs) and RAW 264.7 macrophages, even at concentrations up to 1600 μg/mL [41]. Conversely, *Echinacea* root hexane extract, rich in alkamide and polyacetylene compounds, exhibited cytotoxic effects on human cancer cells through apoptosis induction [42].

Echinacoside has shown to be both safe and non-toxic; however, it does not stimulate cell proliferation. This suggests that the proliferative effect of EAR on 3T3-L1 cells may result from the synergistic action of multiple compounds present in the extract.

H_2_O_2_ is known to induce oxidative stress and is often used to evaluate the antioxidant activity of extracts in cell cultures. *Echinacea* spp. have demonstrated significant biological and pharmaceutical potential [13] due to their rich phytochemical composition. Historically, these plants have been widely used as phytotherapeutic remedies to promote wound healing. Cell proliferation and migration are two features of the wound-healing process. In our study, EAR significantly increased the migration rate of 3T3-L1 cells in a dose-dependent manner, achieving better results than those obtained from echinacoside and allantoin. These findings align with previous studies. Speroni et al. [22] applied *E. pallida* extract topically to rats, analyzing tissue samples after 24, 48, and 72 h. Their results confirmed the anti-inflammatory and wound-healing capacity of *E. pallida* extract and its components, such as echinacoside [22]. Similarly, *E. purpurea* was found to accelerate wound healing in rats following subcutaneous arsenic injection, which had caused extensive skin necrosis [43]. Disruption of the cell monolayer due to scratching results in a loss of cell–cell contact, triggering the release of signaling molecules such as cytokines and growth factors at the wound site. Among these, TNF-*α* is rapidly secreted by fibroblasts in the injured area, initiating the inflammatory phase of wound healing [44]. EAR demonstrated anti-inflammatory effects by reducing the expression of key cytokines, including TNF-*α*, IL-1*β,* and IL-6. Additionally, ROS levels are particularly elevated in the presence of tissue damage, and the observed reduction in ROS suggests that *Echinacea angustifolia* extract may be a promising candidate for wound repair. Indeed, its ability to promote wound healing appears to be linked to its capacity to mitigate both inflammation and oxidative stress.

For optimal wound healing, ECM remodeling is essential, and matrix metalloproteinases (MMPs) play a pivotal role in this process [45]. MMPs are endopeptidases responsible for degrading damaged proteins and forming a temporary ECM during the inflammatory phase. While MMPs are typically expressed at baseline levels, their production increases when tissue remodeling is required. However, excessive MMP activity can lead to cellular dysregulation, potentially impairing wound healing [46]. Our findings indicate that the extract facilitated and accelerated wound healing by modulating MMP-9 production through the NF-κB signaling pathway in fibroblasts. NF-κB is a key regulator of inflammation and immune response and is directly linked to MMP-9 expression, as the promoter region of the MMP-9 gene contains consensus binding sites for NF-κB [47].

Furthermore, excessive ROS levels activate NF-κB, leading to increased MMP-9 expression and delayed wound healing [48]. Among the various molecules involved in wound healing, transforming growth factor (TGF-*β*) proteins are crucial regulators of ECM synthesis and remodeling. Of the three known isoforms, TGF-*β*1 is the most extensively studied. Upon binding to its receptor, TGF-*β*1 stimulates the production of ECM components such as collagen, fibronectin, and hyaluronic acid [49]. Supporting our findings, a recent study reported that treatment with *Echinacea purpurea* powder significantly reduced the wound size in rats, accompanied by an increase in TGF-*β*1 expression [50]. Additionally, ethanol extracts from E. purpurea roots and aerial parts have been shown to inhibit fibroblast-induced collagen contraction [18].

The wound-healing properties of *Echinacea* species have also been attributed to their polysaccharide components, which inhibit hyaluronidase activity [18]. Hyaluronidase inhibition increases hyaluronic acid levels in the ECM, promoting wound healing and reducing local inflammation. The anti-hyaluronidase activity of *Echinacea* species has been associated with echinacoside and caffeic acid [51,52].

Moreover, a previous study reported tyrosinase inhibition of chicoric acid extracted from *E. purpurea* flowers [53], showing an inhibition rate of 89.79% at 20 mg/mL (standard substance). At the same concentration*, E. purpurea* extract exhibited 78.25% inhibition. Echinacoside, abundant in *E. angustifolia*, reported no tyrosinase inhibition at the tested concentrations (1.25–0.04 mM), which could explain the low activity of this extract. Tyrosinase is a rate-limiting enzyme in melanin synthesis in melanocytes, catalyzing the conversion of tyrosine to DOPA and, subsequently, to DOPA chrome, leading to melanin formation. Tyrosinase inhibitors are of interest for cosmetic applications and the treatment of skin pigmentation disorders. In this context, the low anti-tyrosinase activity of *E. angustifolia* could be beneficial following wound healing, as it would help prevent excessive skin bleaching in the scar area.

## 4. Materials and Methods

### 4.1. Chemicals and Reagents

*β*-carotene (CID: 5280489), Folin–Ciocalteu (MDL number: MFCD00132625), 2,2-diphenyl-1-picrylhydrazyl (DPPH) (CID: 86650676), Tween 20 (CID: 443314), linoleic acid (CID: 5280450), tyrosinase from mushroom (CAS Number: 9002-10-2), Levodopa (*L*-DOPA) (CID: 6047), Dulbecco’s modified Eagle’s medium (DMEM) (SID: 56312060), Fetal Bovine Serum (FBS) (MDL number: MFCD00132239), penicillin–streptomycin (CID: 131715954), calcein acetoxymethyl (Calcein-AM) (CID: 390986), hydrogen peroxide (H_2_O_2_) (CID: 784), and all standards used in the study, such as 6-hydroxy-2-5-7-8-tetramethylchroman-2-carboxylic acid (Trolox) (CID: 40634), kojic acid (CID: 3840), gallic acid (CID: 370), butylhydroxytoluene (BHT) (CID: 31404), allantoin (CID: 204), echinacoside (CID: 5281771), caftaric acid (CID: 6440397), chlorogenic acid (CID: 1794427), caffeic acid (CID: 689043), ferulic acid (CID: 445858), chicoric acid (CID: 5281764), verbascoside (CID: 5281800), 3,4 di-caffeoylquinic acid (CID: 5281780), and 3,5 di-caffeoylquinic acid (CID: 13604688) were purchased from Sigma-Aldrich, Milan (Italy).

### 4.2. Plant Extraction

Dried roots of *E. angustifolia* were provided by Farmalabor (Italy, Batch Nr. P1403826-000), and 10 g was extracted by 85% ethanol solution (50 mL) for 2h under stirring. The extract was filtered by filter paper under vacuum; then, the solvent was removed by rotary evaporator to obtain the dried crude extract of *E. angustifolia* roots (EAR). The extract was left in the dark at room temperature until its use. The plant name was checked with http://www.theplantlist.org, accessed on 22 May 2021.

### 4.3. Qualitative and Quantitative Analysis by HPLC-DAD

Qualitative and quantitative investigation of phenolic compounds from the EAR extract was conducted using high performance liquid chromatography (HPLC), and the identification was performed by comparison with the retention time and relative UV–Vis spectra (200–400 nm) of the standards, as previously reported [32].

The EAR extract was solubilized in methanol (50 mg/mL) and filtered by polytetrafluoroethylene (PTFE) syringe filter (0.20 μm) for reverse-phase HPLC analysis (Gilson, Villers le Bel, France) equipped with a Diode Array Detector (DAD-Gilson). The experiments were carried out on a Spherisorb ODS2 C18 column (25.00 cm × 0.46 mm × 5.00 μm; Waters, Milford, MA, USA); the temperature of the thermostat Jetstream 2 Plus (Thermotechnic Products, Langenzersdorf, Austria) was set at 25 °C. The mobile phase was as follows: A 0.1% *O*-phosphoric and B acetonitrile (Merck KGaA, Darmstadt, Germany). Elution was performed by using a flow rate of 1.5 mL min^−1^ and following the gradient elution program, as reported by previous study [32]. The injection volume was set to 20 μL.

### 4.4. Qualitative Analysis by UHPLC-DAD-ESI-Orbitrap

For LC-MS analysis, the EAR extract was solubilized in methanol and 5% of DMSO (5 mg/mL) and filtered by cellulose acetate (CA) syringe filter (0.20 μm). The sample was analyzed using the UHPLC-DAD-ESI-Orbitrap Exploris™ 120 Mass Spectrometer (Thermo Scientific, Bremen, Germany), and the compounds were identified by considering the retention time and fragmentation pattern of the corresponding standard compounds. The UHPLC-DAD analysis was conducted using a reversed-phase system (Vanquish UHPLC Thermo Scientific system coupled to DAD and Orbitrap Exploris™ 120 Mass Spectrometer), equipped with a binary pump. The column used was a Phenomenex (1.6 μm PS C18 100 Å 100 × 2.1 mm). As mobile phases, two different solvents were used: water + 0.1% formic acid (A) and MeOH + 0.1% formic acid (B), programmed in the following gradient: 0–5 min 5–35%B, 5–25 min 35–100%B; 25–35 min 100%B. Before use, the mobile phases were filtered through a 0.20 μm membrane. The flow rate was 0.2 mL/min, and the time analysis was 35 min. The injected sample volume was 5 μL. The column temperature was set at 30 °C. Electrospray mass spectra data were acquired in negative ionization mode with a voltage set at 3500 V, maintaining a resolution of 120,000 both in full MS and dd-MS2 scans and across an *m*/*z* range of 80–1200. Nitrogen was used as the collision gas, and the collision-induced fragmentation (CID) of the analytes was achieved using 30 eV energy. The ion transfer tube and vaporizer temperatures were 320 °C and 280 °C, respectively. The sheath gas, Aux gas, and sweep gas were 40, 20, and 0, respectively. EASY-ICTM was used as the internal mass calibration. The multiple reaction monitoring (MRM) quantitative method was developed for standard compounds, and the analysis of the data was carried out using Chromeleon 7.3.1 Software (Thermo Scientific™ Dionex™ U.S. Patents). Caftaric acid, chlorogenic acid, caffeic acid, echinacoside, ferulic acid, and verbascoside standards were solubilized in MeOH + 5% DMSO at different range concentrations between 0.001 and 100 ppm. Two biological replicates were prepared, and triplicate technical replicates were conducted for each biological replicate. All the standards were filtered using 0.22 μm CA filters before injection.

### 4.5. Polysaccharide Content

The polysaccharide content was determined, as previously reported, by a de-proteinization step with chloroform–*n*-butanol and then by precipitation of polysaccharides in ethanol [32]. The measurement of the polysaccharide concentration was determined with phenol–sulfuric acid assay by measuring the absorbance at 490 nm after 1 h. Distilled water was used a negative control.

### 4.6. Total Polyphenolic Content (TPC) and Antioxidant Activity

The TPC was measured by Folin–Ciocalteu reagent [54]. Briefly, the EAR extract (75 μL), dissolved in methanol, was added to the Folin–Ciocalteu reagent (500 μL) and Na_2_CO_3_ (500 μL). Distilled water was added to the test solution reaching a final volume of 1.5 mL. The absorbance recorded at 723 nm was used to quantify the polyphenol content by the gallic acid standard curve (mg of gallic acid equivalents per g of crude extract, mg GAE/g).

#### 4.6.1. DPPH-Scavenging Activity

The radical scavenging ability of the EAR extract was determined by DPPH assay. The extract (50 μL) at different concentrations was added to the DPPH solution (200 μL, 100 μM); the absorbance (515 nm) was read after 30 min of incubation in the dark [55,56].

#### 4.6.2. Lipid Peroxidation Inhibition

The inhibition of lipid peroxidation was carried out by *beta*-carotene bleaching (BCB) assay as previously reported [57].

The *β*-carotene/linoleic acid emulsion (950 μL) was added to the extract (50 μL) to reach a final concentration of 0.1 mg/mL. Aliquots of 250 μL were transferred to the 96-multiwell and incubated at 50 °C for 3 h. The absorbance was read at 470 nm, and the results were expressed as % antioxidant activity (A.A.).

#### 4.6.3. In Vitro Tyrosinase Inhibition Assay

The tyrosinase inhibition was carried out by the *L*-DOPA in vitro test [58]. Phosphate buffer (125 μL, 50 mM, pH 6.8), was added to the standard or extracts at different concentrations (25 μL) and to the tyrosinase enzyme (50 μL, 50 U/mL) and incubated at 37 °C for 15 min. The reaction was monitored for 10 min at 475 nm after the addition of the *L*-DOPA substrate (50 μL). Kojic acid was used as the positive control. The experiment was performed in triplicate, and the IC_50_ value was determined by interpolation with concentration–response curves.

### 4.7. Cell Culture Maintenance

The mouse embryonic fibroblast cell line 3T3-L1 (American Type Culture Collection, CL-173, LGC Standards S.r.l. Sesto San Giovanni, Italy) was maintained in DMEM at 37 °C in humidified atmosphere containing 5% CO_2_ and supplemented with 10% FBS, 100 IU/mL penicillin, and 100 μg/mL streptomycin.

### 4.8. Cell Proliferation and Viability Assay Using 3T3-L1 Cell Line

3T3-L1 cells (1 × 10^4^ cells/well) were seeded into 96-well black plates and synchronized for 3 h with 1% FBS medium. Then, cells were exposed to different concentrations of extract (1000, 100, 50, 10, and 5 μg/mL), echinacoside (0.50, 0.25, 0.10, 0.05, and 0.01 mM), allantoin (0.50, 0.25, 0.10, 0.05, and 0.01 mM), or the vehicle (culture medium) as the negative control, at 37 °C for 24 and 48 h. The medium was replaced with 100 μL of 1 μM calcein-AM (Life Technologies Corporation, Segrate, MI, Italy) in PBS and incubated for 30 min at 37 °C before the detection of the fluorescence by using FLEXSTATION 3 (Ex 490 nm, Em 510–570 nm) [57].

### 4.9. Hydrogen Peroxide-Induced Oxidative Stress Using 3T3-L1 Cell Line

H_2_O_2_ was used to generate oxidative stress in 3T3-L1 cells. The dose of H_2_O_2_ that reduces cell viability by 80% after 24h was used for the assay. The cells were seeded into 96-well black plates (1 × 10^4^ cells/well) and treated with different concentrations of H_2_O_2_ (10, 5, 1, 0.50, 0.25, 0.13, and 0.06 mM). The cell viability was measured by the calcein-AM assay. The dose chosen was 5 mM and was used for further analysis. 3T3-L1 cells were treated with different concentrations of extract or echinacoside as follows: (a) cells were treated with H_2_O_2_ 5 mM for 3 h and then 24 h with the extract, echinacoside, or medium; (b) cells were treated for 24 h with extract, echinacoside, or medium and then with H_2_O_2_ 5 mM for 3 h; (c) cells were exposed concomitantly to extract, echinacoside, or medium and H_2_O_2_ 5 mM for 24 h. The viability was performed using calcein-AM, as mentioned above [31].

### 4.10. Determination of Migration Using Wound Healing Assay

The ability of the *E. angustifolia* extract, echinacoside, and allantoin compounds to modulate the cell migration process was determined by an in vitro wound-healing migration assay [31]. For the migration assay, 3T3-L1 cells were seeded in 35 mm cell culture dishes (Cellview cell culture dish, 35/10mm glass bottom, Greiner Bio-one) at the density 1 × 10^6^ cells/dish until a confluent cell monolayer was reached. Linear wounds were generated by using a sterile 10 μL plastic pipette tip. Cellular debris were removed by washing with PBS and replaced with 2 mL of DMEM 1% FBS containing *E. angustifolia* crude extract (100 μg/mL, 50 μg/mL, and 10 μg/mL), echinacoside (100 μM), and allantoin (100 μM); DMEM 1% FBS was used control. Time-lapse images were obtained by BioStation IM incubator with a camera. The acquired images were analyzed using ImageJ 1.46 software, and the results of the migration rate were reported as μm/h as follows.

(1) $$Migration\;rate\;(\%)=\frac{distance\;within\;scratch\;(t0)-distance\;within\;scratch\;(tn)}{distance\;within\;scratch\;(t0)},$$

where *n* = every 20 min.

### 4.11. Assessment of Scratch Stress-Induced Intracellular ROS in 3T3-L1 Cells

The intracellular reactive oxygen species (ROS) levels were measured by DCFH-DA. 3T3-L1 cells were plated at a density of 1 × 10^5^ cells/well in a 24-well plate and incubated until 100% confluence. After that, the cells were scratched and treated with EAR (100 μg/mL), allantoin (ALL, 100 μM), and echinacoside (ECH, 100 μM) for 6 h. Finally, the cells were stained with 10 μM DCFH-DA for 30 min at 37 °C in the dark, and the fluorescence was measured by BD FACSCanto II (BD Pharmingen, San Jose, CA, USA) (λex 485 nm and λem 515–540 nm).

### 4.12. Quantitative RT-PCR

3T3-L1 cells were treated with EAR (100 μg/mL), allantoin (ALL, 100 μM), and echinacoside (ECH, 100 μM) for 6 h after scratching. RNA was extracted using a specific kit (Qiagen, Hilden, Germany) and then was transcribed to cDNA using random primers and a High-Capacity cDNA Reverse Transcription Kit (ThermoFisher Scientific, Waltham, MA, USA, Life Technologies Corporation, Carlsbad, CA, USA). The cDNA was amplified via real-time PCR using iTaqTM Universal SYBR^®^ Green Supermix (Bio-Rad, Milan, Italy) by the 7500 Fast Real-Time PCR System (Applied Biosystems, Foster City, CA, USA). Primers were designed for spanning exon–exon junctions, eliminating undesirable genomic DNA amplification. The comparative threshold cycle method (ΔΔCt) was used to quantify the relative amounts of product transcripts, with Polymerase II as the housekeeping gene. The specificity of amplicons was confirmed by melting-curve analysis. The primer sequences are *polymerase II* (F: 5′-GAC AAC GAG GAC AAT TTC GAC G-3′; R: 5′-GGA GAA TCT CGA CAT TTT CCT GG-3′); *TNF-α* (F: 5′-CTG AAC TTC GGG GTG ATC GG-3′; R: 5′-GGC TTG TCA CTC GAA TTT TGA GA-3′); *IL-1β* (F: 5′-GAA ATG CCA CCT TTT GAC AGTG-3′; R: 5′-TGG ATG CTC TCA TCA GGA CAG-3′); *MMP-9* (F: 5′-CTG GAC AGC CAG ACA CTA AAG-3′; R: 5′-CTC GCG GCA AGT CTT CAG AG-3′).

### 4.13. Western Blotting

3T3-L1 cells were seeded in 12-well plates (1.5 × 10^5^ cells) and treated with EAR (100 μg/mL), ALL (100 μM), and ECH (100 μM) for 6 h after cell scratching. The cells were washed with ice-cold PBS and lysed in RIPA buffer supplemented with a protease inhibitor cocktail. After centrifugation at 13,000× *g* for 10 min, the supernatant was prepared as a protein extract. Protein concentrations were determined using the Bradford assay. Equal amounts of protein from each sample were mixed with a sample buffer and separated using Bolt™ 4–12% Bis- Tris Plus gels (Thermo Fisher Scientific, Monza, Italy). The separated proteins were transferred to a nitrocellulose membrane. The membranes were blocked with 5% (*w*/*v*) non-fat dried milk and 0.025% Tween-20 in PBS (PBS-T) and incubated for 1 h at room temperature. Then, the membranes were probed overnight at 4 ◦C with specific primary antibodies: (1:1000 anti-NF-*k*B; 1:1000 anti-TGF-*β-*1; 1:1000 MMP9 and 1:5000 anti-*β*-actin). After incubation, membranes were washed three times with PBS-T (PBS containing 0.1% Tween 20) for 10 min and incubated with the appropriate horseradish peroxidase-conjugated secondary antibody at room temperature for 1 h; signals were visualized by Super Signal West Femto Maximum Sensitivity Substrate (Thermo Fisher Scientific, Monza, Italy), using the Chemidoc XRS detection system equipped with Image Lab Software (BioRad, version 5.2.1) for image acquisition. The quantification of proteins bands was achieved by determination of the relative optical density using ImageJ software (version 1.53k, National Institute of Health, Bethesda, MD, USA).

### 4.14. Statistical Analysis

All experiments were performed in triplicate. The results are expressed as the mean ± standard deviation (SD). GraphPad Prism 5 Software, Inc. (San Diego, CA, USA) was used for statistical analysis by Tukey test, and a *p* value of 0.05 or less was considered statistically significant. Spectrophotometric determinations were carried out using SPECTROstar^Nano^ (BMG Labtech, Ortenberg, Germany).

## 5. Conclusions

*E. angustifolia* extract demonstrated in vitro antioxidant and anti-inflammatory activity and can be considered a proliferation and migration enhancer in the 3T3-L1 cell line. In addition, the cytoprotective effect on 3T3-L1 cells against hydrogen peroxide can be attributed to its phenolic composition. The echinacoside represents 83% of the compounds identified in the extract by quantitative HPLC analysis, followed by ferulic acid, chlorogenic acid, and caftaric acid.

While echinacoside was found to be safe, non-toxic, and effective in preventing oxidative cell damage and reducing inflammation by suppressing proinflammatory cytokines, it was ineffective in stimulating cell proliferation. These findings suggest that EAR’s proliferative effect on 3T3-L1 cells is likely due to the synergistic interaction of multiple compounds rather than a single active molecule. Further investigation should focus on identifying unknown compounds and in vivo studies investigating antioxidant enzymes to understand the molecular mechanisms involved in the wound-healing process of *E. angustifolia*.

## Figures and Tables

**Table 1:** Characterization of *E. angustifolia* root extract phytochemicals by liquid chromatography–tandem mass spectrometry.

Pk No.	RT (min)	[M-H]^−^ *m/z* Calculated	[M-H]^−^ *m/z* Observed	Predicted Molecular Formula	MS/MS *m/z*	Compound Identity	References
1	9.58	311.0409	311.0407	C_13_H_12_O_9_	179, 149 (100), 135	Caftaric acid	[24]
2	10.66	353.0878	353.0874	C_16_H_18_O_9_	191 (100)	Chlorogenic acid	[25]
3	11.04	179.0350	179.0349	C_9_H_8_O_4_	135 (100)	Caffeic acid	[26]
4	11.46	785.2510	785.2498	C_35_H_46_O_20_	623, 161 (100)	Echinacoside	[27]
5	12.72	193.0506	193.0507	C_10_H_10_O_4_	178, 134 (100)	Ferulic acid	[28]
6	12.91	623.1981	623.1980	C_29_H_36_O_15_	461, 161 (100)	Verbascoside	[29]

## Data Availability

All data presented in this study are available upon request from the corresponding author.
